# Identification and Validation of Afatinib Potential Drug Resistance Gene *BIRC5* in Non-Small Cell Lung Cancer

**DOI:** 10.3389/fonc.2021.763035

**Published:** 2021-11-03

**Authors:** Xiaoxi Zhu, Renyu Zhou, Yuanzhi Lu, Ying Zhang, Qiang Chen, Yin Li

**Affiliations:** ^1^ Department of Oncology, First Affiliated Hospital of Jinan University, Guangzhou, China; ^2^ Department of Clinical Pathology, First Affiliated Hospital of Jinan University, Guangzhou, China

**Keywords:** bioinformatic analysis, NSCLC, afatinib resistance, BIRC5, TKI

## Abstract

**Introduction:**

Resistance to second-generation epidermal growth factor receptor-tyrosine kinase inhibitor (EGFR-TKI), afatinib, is the most significant challenge in the clinical management of non-small cell lung cancer (NSCLC), and the underlying mechanisms remain unclear.

**Methods:**

Genomic signatures that may confer afatinib resistance in NSCLC were identified *via* data mining of public databases and integrative bioinformatic analyses. Furthermore, acquired afatinib-resistant lung adenocarcinoma cell lines (HCC827 AR) were established by long-term exposure under afatinib *in vitro* for stepwise escalation. The expression of baculovirus IAP repeat protein 5 (BIRC5) was detected by western blot, and cellular viability of HCC827 AR was determined by CCK8.

**Results:**

Through integrative bioinformatic analyses of public datasets, overexpression of baculovirus IAP repeat protein 5 (BIRC5) was identified in both afatinib-resistant NSCLC cells and tissues, and BIRC5 overexpression was positively correlated with lymph node metastasis as well as pathological stage in NSCLC. Furthermore, NSCLC patients with BIRC5 overexpression showed poor survival outcomes. Immune infiltration analysis suggested that BIRC5 expression was significantly inversely correlated with tumor-infiltrating cell numbers and immune biomarker expression in NSCLC. The functions of genes co-expressed with BIRC5 were mainly enriched in cell cycle mitotic phase transition, double-strand break repair, and negative regulation of the cell cycle process signaling pathway. In addition, overexpression of BIRC5 protein was detected in afatinib-resistant cells by western blot, while BIRC5-expressing cells treated with BIRC5 inhibitor, YM155, were sensitive to afatinib.

**Conclusions:**

In this study, we showed that overexpression of BIRC5 resulted in resistance to afatinib in NSCLC and BIRC5-specific inhibitors may overcome the resistant phenotype, indicating that dysregulation of the apoptotic cell death pathway may be the key mechanism underlying TKI resistance in the development of NSCLC.

## Introduction

Non-small-cell lung cancer (NSCLC) is the most prevalent subtype of lung cancer (approximately 85%) (1); furthermore, it is mostly metastatic at diagnosis and represents the leading cause of cancer death worldwide (2). Most patients are diagnosed at an advanced stage (3, 4). The discovery of activating mutations in epidermal growth factor receptor (EGFR)and their use as predictive biomarkers to tailor patient therapy with EGFR TKIs has revolutionized the treatment of patients with advanced EGFR-mutant NSCLC (5).

Afatinib is an irreversibly mutant EGFR-TKI and the first-line FDA-approved treatment for locally advanced or metastatic NSCLC (6). While afatinib achieves superior efficacy in progression-free survival (PFS) and overall survival (OS) compared with conventional chemotherapy in NSCLC, progression inevitably occurs after EGFR TKI treatment for acquired resistance, which presents challenges in the treatment of NSCLC (7, 8). The mechanisms of acquired resistance are classified into three types: acquired mutation of targetable driver genes, bypass of signaling pathway activation, and histological lineage-transformation (9). Accordingly, pharmacological interception of the propensity of tumor cells to bypass signaling pathways derails their signaling or adhesion receptors and may allow the identification of novel targets for cancer therapy (10). An understanding of the mechanistic bases for drug resistance would continue to inform the development of strategies to overcome or prevent clinical acquired resistance, thereby providing greater therapeutic benefits for cancer patients (11).

BIRC5 (also known as survivin) is a small protein belonging to the inhibitor of apoptosis protein family that inhibits caspases and blocks cell death. It is abundantly expressed in tumors compared with adult differentiated tissues and is associated with poor prognosis in many human neoplasms (12). Because of selective expression in tumor but not normal tissues, for over a decade, BIRC5 has drawn considerable attention as a potential novel drug target in a variety of human cancers and has consistently been demonstrated to be a critical factor in tumor progression (13). Most studies of BIRC5 have focused on sensitization to chemotherapy and radiotherapy, while the level of heterogeneity among patients receiving targeted drug treatment and its biological significance have not yet been comprehensively investigated.

In this work, BIRC5 was initially identified as a potential candidate in the Gene Expression Omnibus (GEO) database. Then, we comprehensively searched the dataset and conducted a systematic bioinformatic analysis of potential genes promoting afatinib resistance in NSCLC. Moreover, the afatinib resistance role of BIRC5 was further validated by combining multiple tools, including protein/gene interactions(PPI), biological process annotation, and prediction of resistance mechanisms. Our study indicates a potential target and associated mechanisms of afatinib resistance, and suggests that BIRC5 could be a prognostic biomarker for afatinib treatment.

## Materials and Methods

### Microarray Data

The GEO database is a high-throughput microarray and sequence functional genomic database (https://www.ncbi.nlm.nih). In this study, the GSE62504 dataset consisted of two afatinib-resistant HCC827 replicates and one parental HCC827 cell line (14). The GSE75037 dataset (15) included 83 lung adenocarcinoma (LUAD) samples and 83 matched adjacent lung samples, whereas the GSE18842 dataset contained 32 squamous cell carcinoma, 14 adenocarcinoma, and 45 adjacent lung tissues (16).

### Processing of Microarray Data

The original microarray data files of these three downloaded datasets were analyzed through GEO2R (https://www.ncbi.nlm.nih.gov/geo/geo2r/). This online tool can be used to compare two groups under the same experimental settings or more sets of samples (17). The P<0.05 and |fold change (FC)| > 1.0 were set as the cutoff standards to define the DEGs.

### Functional Annotation and Pathway Enrichment Analysis

To perform functional annotation of DEGs, we applied annotation, visualization, and used a comprehensive database (DAVID, https://david.abccncifcrf.gov/) (18) to perform the Gene Ontology (GO) and Kyoto Encyclopedia of Genes and Genomes (KEGG) analyses, specifically GO enrichment analysis and KEGG pathway analysis.

### ONCOMINE

The gene expression array dataset of ONCOMINE (www.oncomine.org) is a publicly accessible, online cancer microarray database that helps facilitate research using genome-wide expression analyses (19). For DEGs, comparison between cancer specimens and normal control dataset analysis was performed.

### TIMER Analysis

The TIMER database was used to systematically analyze tumor-infiltrating immune cells (TIICs) in 32 cancer types using more than 10,000 samples from The Cancer Genome Atlas (TCGA) (https://cistrome.shinyapps.io/timer/) database (20). We initially used this database to assess differences in DEG expression levels in tumor types and explored the relationship between the expression of BIRC5 and the abundance of immune infiltrates in lung squamous cell carcinoma (LUSC) and LUAD by considering P<0.05 as the cut-off criterion for statistical significance.

### UALCAN

The platform UALCAN is an omnibus and interactive web-based tool based on The Cancer Genome Atlas (TCGA) for deep analysis of gene expression using genomics data from 31 cancer types (21). The database UALCAN was used to analyze the correlation between BIRC5 mRNA transcriptional levels in LUSC and LUAD patients with different stage, gender, age, smoking habits, and lymph node metastasis. P<0.05 was regarded as indicating statistically significant results.

### Kaplan-Meier Plotter

Kaplan-Meier plotter (https://www.kmplot.com) is an online survival analysis tool consisting of 10,461 cancer samples (including samples from 5143 breast cancer, 1816 ovarian cancer, 2437 lung cancer, and 1065 gastric cancer patients) that can facilitate evaluation of the impact of 54,675 gene pairs on overall survival OS (22). Based on the expression levels of DEGs, lung cancer patients were divided into a high expression group and a low expression group, the first progression(FP), OS and post progression survival (PPS) rate was further analyzed.

### LinkedOmics

The LinkedOmics database contains multi-omics data and clinical data for 32 cancer types and 11,158 patients from the TCGA project (23). The “LinkInterpreter” module was used to derive biological insights and perform analysis of kinase targets, miRNA targets, and transcription factor targets for BIRC5 chemokines. Gene Set Enrichment Analysis (GSEA) was used to perform analyses with a minimum number of genes (size) of 3 and a simulation of 500 within the LUAD dataset. Results were analyzed statistically using the Pearson’s correlation test. The P value cutoff was 0.05.

### GeneMANIA

GeneMANIA (https://www.genemania.org/) was used to predict the potential function of the BIRC5 gene and to predict the functions of specific genes (24). After obtained the kinase networks using LinkedOmics, we put those genes into GeneMANIA and understood their potential functions.

### GSCALite

GSCALite, a bioinformatics platform for gene set cancer analysis, offered several type of analyses, including methylation analysis, cancer-related pathway analysis, miRNA network analysis, etc. (25). GSCALite provided pathway activity analysis and drug sensitivity analysis in our study with TCGA SKCM sample. The spearman correlation was used to explore the correlation between the gene expression and drug sensitivity.

### Cell Lines and Cell Culture

Afatinib-sensitive NSCLC cell lines with mutated EGFR (DelE746-A750), HCC827 (Keygenbio, Nanjing, China) were cultured in RPMI-1640 medium (Gibco, USA) supplemented with 10% fetal bovine serum (Gemini, USA), penicillin (100 U/mL) and streptomycin (100 mg/mL). All cell lines were maintained in a humidified incubator at 37°C with 5% CO_2_.

### Establishment of the Drug-Resistant Cell Line

The HCC827 cells were cultured in a medium containing afatinib (Selleck Chemicals, Houston, TX, USA)at 1 nmol/L. When the cells showed viability similar to that of cells without afatinib, we gradually increased the concentration of afatinib until it reached 6 μmol/L. The entire induction period lasted approximately six months.

### Cell Counting Kit-8

HCC827 and HCC827-AR cells (3×10^3^) were seeded in 96-well plates, grown overnight, and then treated with varying drug concentrations for 72 h; then, serum-free medium was replaced. Next, 10 μL of CCK8 (Beyotime, Shanghai, China) was added to each well for incubation at 37°C with 5% CO_2_ for 2 h. The OD value was measured at 450 nm wavelength (Thermo, Waltham, MA, USA).

### Western Blot

The cells were lysed with RIPA buffer (Beyotime, Beijing, China) in the presence of protease inhibitor PMSF (Beyotime, Beijing, China). Protein samples were quantified using BCA protein assay kit (Beyotime, Beijing, China). Equal amounts of proteins (25 μg) were separated on 15% SDS-polyacrylamide gels and transferred to polyvinylidene fluoride membranes (Beyotime, Beijing, China). Then, the membrane was blocked with defatted milk at room temperature for 2 h and incubated with anti-BIRC5 rabbit polyclonal antibody (1:500; Cell Signaling Technology, #2808S) and anti-GAPDH rabbit monoclonal antibody (1:6000; Cell Signaling Technology, #5174S) at 4°C overnight. Next, the membrane was incubated with fluorescence-labeled anti-rabbit IgG antibody (1:1000; Cell Signaling Technology, #7074) at room temperature for 2 h and then visualized using enhanced chemiluminescence (Thermo, Waltham, MA, USA).

### Statistical Analysis

GraphPad Prism 8 software (GraphPad Software, Inc., USA) statistical software was used to process the data, and *P*<0.05 was considered to define statistical significance.

## Results

### Identification and Functional Characterization of Upregulated DEGs in Afatinib-Resistant NSCLC Cells

**Figure 1:**
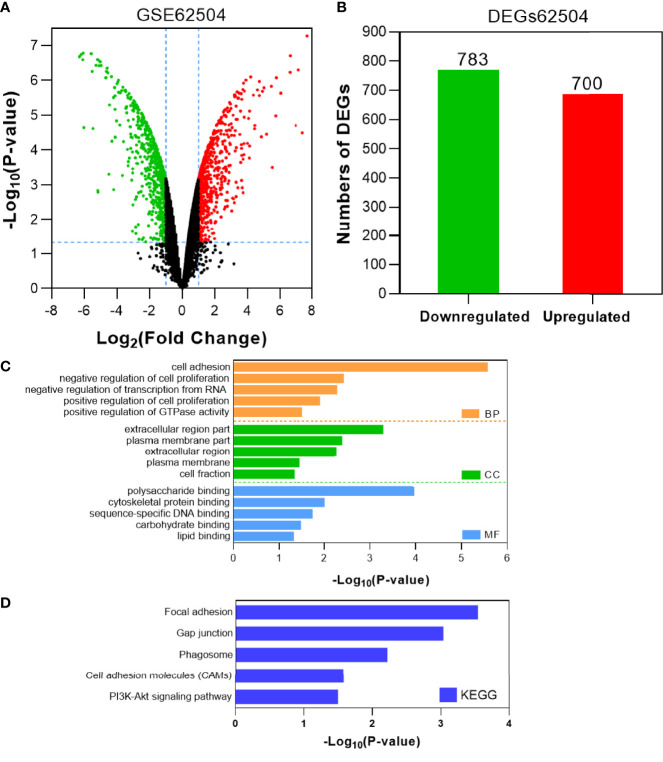
Identification and characterization of DEGs from the GSE62504 dataset. **(A)** Volcano plot of DEGs between afatinib-resistant cells and parental cells. Red dots indicate significantly upregulated DEGs in afatinib-resistant cells; green dots, afatinib-resistant DEGs that were downregulated; black dots, no significant difference (P < 0.05 and |log2FC|>1.0 as the threshold). **(B)** Distribution of DEGs of significance in afatinib-resistant cells. The top five GO terms **(C)** and KEGG enriched pathways **(D)** of significantly upregulated DEGs in NSCLC tissues are indicated. BP, biological process; CC, cell component; MF, molecular function.

To identify the potential genes conferring afatinib resistance in NSCLC, we investigated GSE62504 using |log2 (FC)|>1.0 and *P <*0.05 as thresholds to screen for differential genes (**Figure 1A**). A total of 1483 DEGs were screened, of which 700 DEGs were found to be significantly upregulated and 783 DEGs were downregulated (**Figure 1B**). To characterize the functions of these significantly upregulated DEGs, GO and KEGG analyses were then performed as previously described. The top five GO terms and enrichment pathways were determined, and these significantly upregulated DEGs were found to be highly associated with cell adhesion, inhibition of apoptosis, and inhibitor RNA transcription height (**Figure 1C**). As shown in **Figure 1D**, the DEGs that were simultaneously upregulated were also enriched in intercellular junctions and phagosomes, cell adhesion pathways, and PI3K-Akt signaling pathways.

### BIRC5 Was Identified as the Gene Conferring Afatinib Resistance in NSCLC

**Figure 2:**
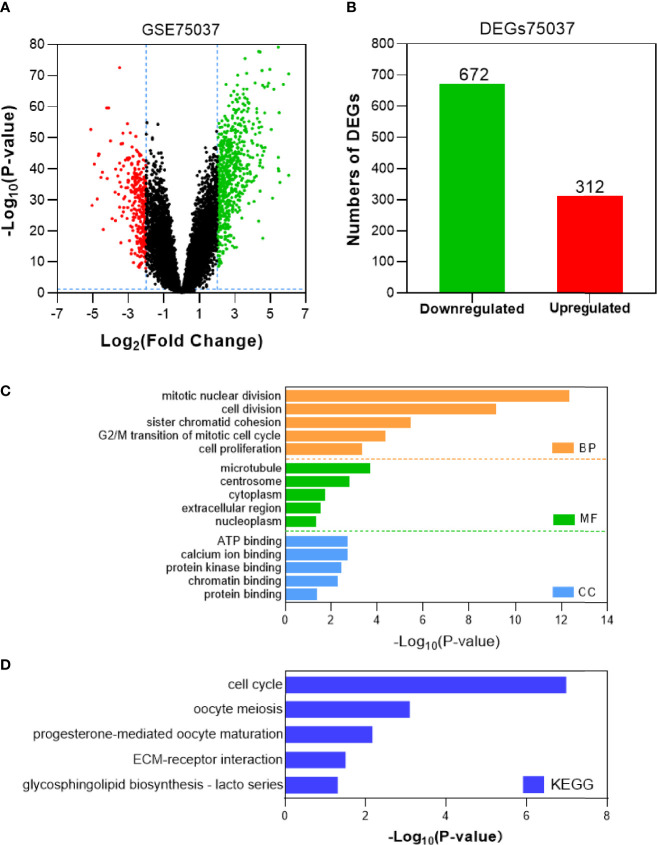
Identification and characterization of DEGs from the GSE75037 dataset. **(A)** Volcano plot of DEGs between NSCLC tissues and adjacent normal tissues. Red dots are significantly upregulated DEGs in NSCLC tissues; green dots, DEGs downregulated in NSCLC tissues; black dots, genes with no significant difference (P<0.05 and |log2FC|>2 as the threshold). **(B)** Distribution of significant DEGs in NSCLC tissues. The top five GO terms **(C)** and KEGG enriched pathways **(D)** of significantly upregulated DEGs in NSCLC tissues are indicated. BP, biological process; CC, cell component; MF, molecular function.

**Figure 3:**
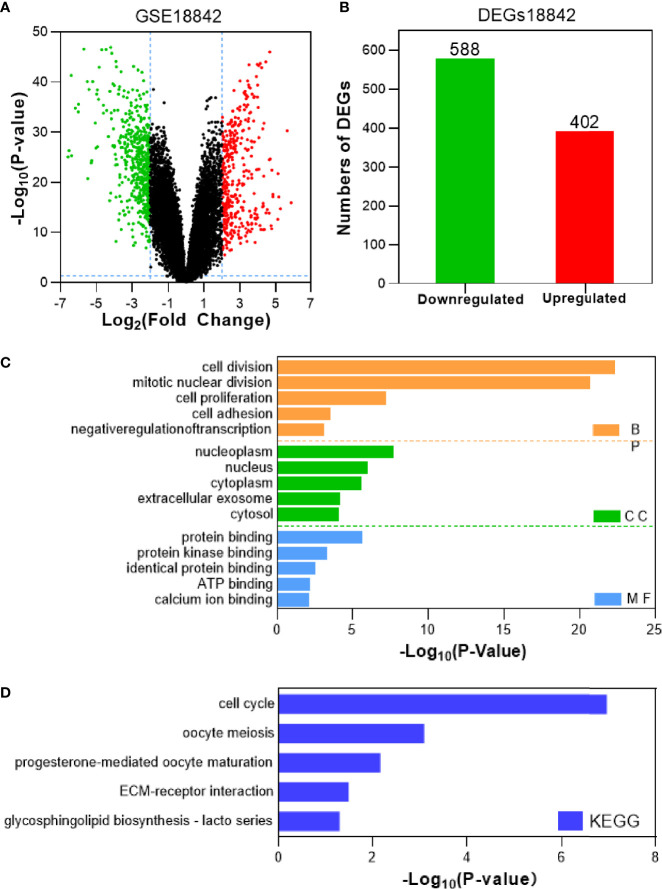
Identification and characterization of DEGs from the GSE18842 dataset. **(A)** Volcano plot of DEGs between NSCLC tissues and adjacent normal tissues. Red dots are significantly upregulated DEGs in NSCLC tissues; green dots, DEGs downregulated in NSCLC tissues; black dots, genes with no significant difference (P<0.05 and |log2FC|>2 as the threshold). **(B)** Distribution of significant DEGs in NSCLC tissues. The top five GO terms **(C)** and KEGG enriched pathways **(D)** of significantly upregulated DEGs in NSCLC tissues are indicated. BP, biological process; CC, cell component; MF, molecular function.

**Figure 4:**
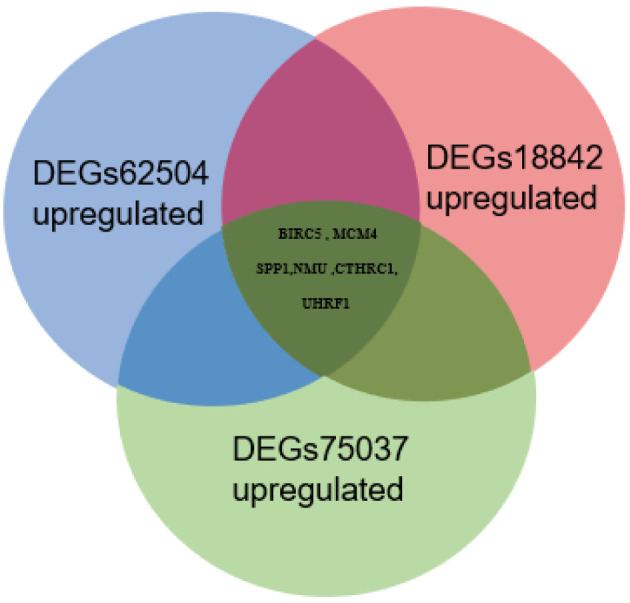
Venn diagram of significantly upregulated DEGs from the GSE62504, GSE75037, and GSE18842 datasets.

Tumor gene heterogeneity is one of the determinants of drug resistance during tumor treatment (26, 27). To screen for genes related to the occurrence of afatinib resistance, the GSE75037 dataset consisting of lung cancer and adjacent normal tissues was further searched through the GEO database. A total of 984 DEGs were screened (**Figure 2A**), of which 312 genes were significantly upregulated and 672 genes were downregulated in cancer tissues (**Figure 2B**). Interference agents with mitotic arrest in cells promotes their death, which is a successful clinical strategy (28). The top GO terms and KEGG pathways of upregulated DEGs affected nuclear division and mitosis (**Figure 2C**) and the cell cycle (**Figure 2D**). Gene expression control mechanisms are complex and unique, and the integration of transcription and proteomic data provides additional information about gene expression control that cannot be obtained from data from a single source (29). To verify the reliability of the results, the GSE18842 datasets composed of data on lung cancer and normal tissues were retrieved. As the results show in **Figure 3A**, 990 DEGs were identified, among which 402 DEGs were up-regulated and 588 DEG were significantly down-regulated (**Figure 3B**). The GO terms were highly consistent with those for the GSE75037 dataset (**Figure 3C**), while the DEGs were enriched in cell cycle pathways (**Figure 3D**). Upon comparing the DEGs significantly upregulated in GSE62504, GSE18842, and GSE75037 (**Figure 4**), six genes were identified, namely BIRC5, MCM4 (30), SPP1 (31), NMU (32), CTHRC1 (33), and UHRF1 (34) had reported on drug resistance. However, little research has explored BIRC5 in the context of afatinib resistance in lung cancer (35). Therefore, we chose BIRC5 as the potential target gene of interest in this study.

### Validation of BIRC5 Related to Afatinib Resistance in NSCLC

**Figure 5:**
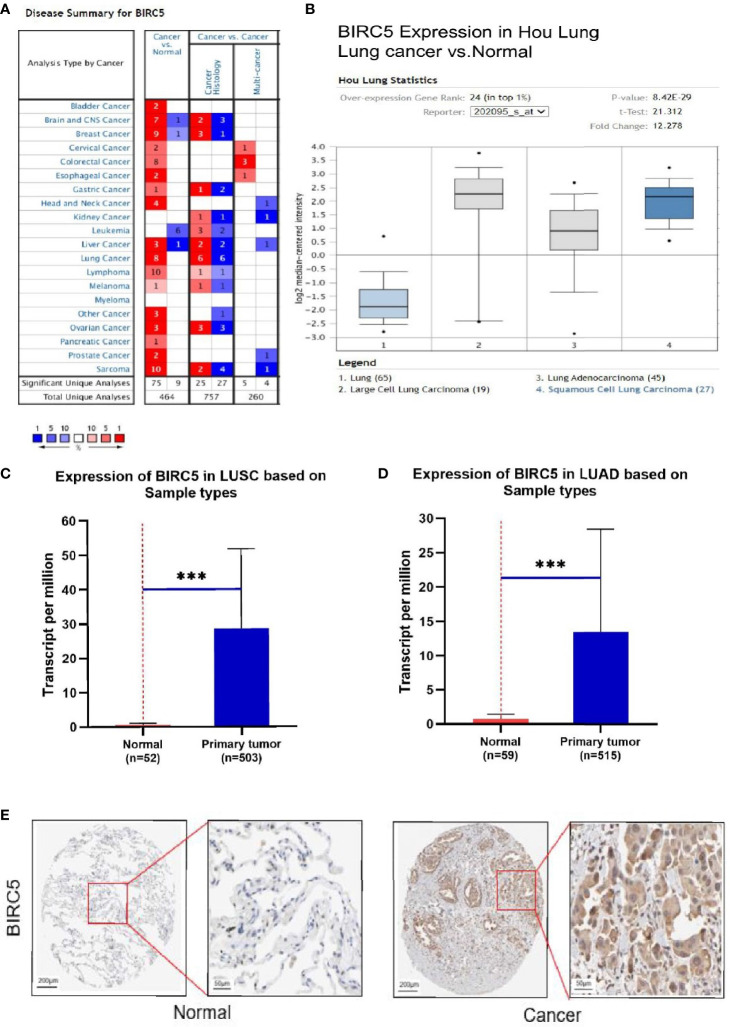
The transcription levels of BIRC5 in lung cancer. **(A)** The expression level of BIRC5 in different cancers compared with that in normal tissues in the Oncomine database (P<0.0001, |FC|>2, and gene ranking of all). Compared with normal samples, BIRC5 mRNA was overexpressed in lung cancer samples in the Oncomine database **(B)** and UALCAN database **(C, D)** ***P < 0.001. **(E)** Immunohistochemical staining of BIRC5 protein expression in normal lung tissue and NSCLC tissue was obtained from the Human Protein Atlas online database (magnification, ×40).

**Figure 6:**
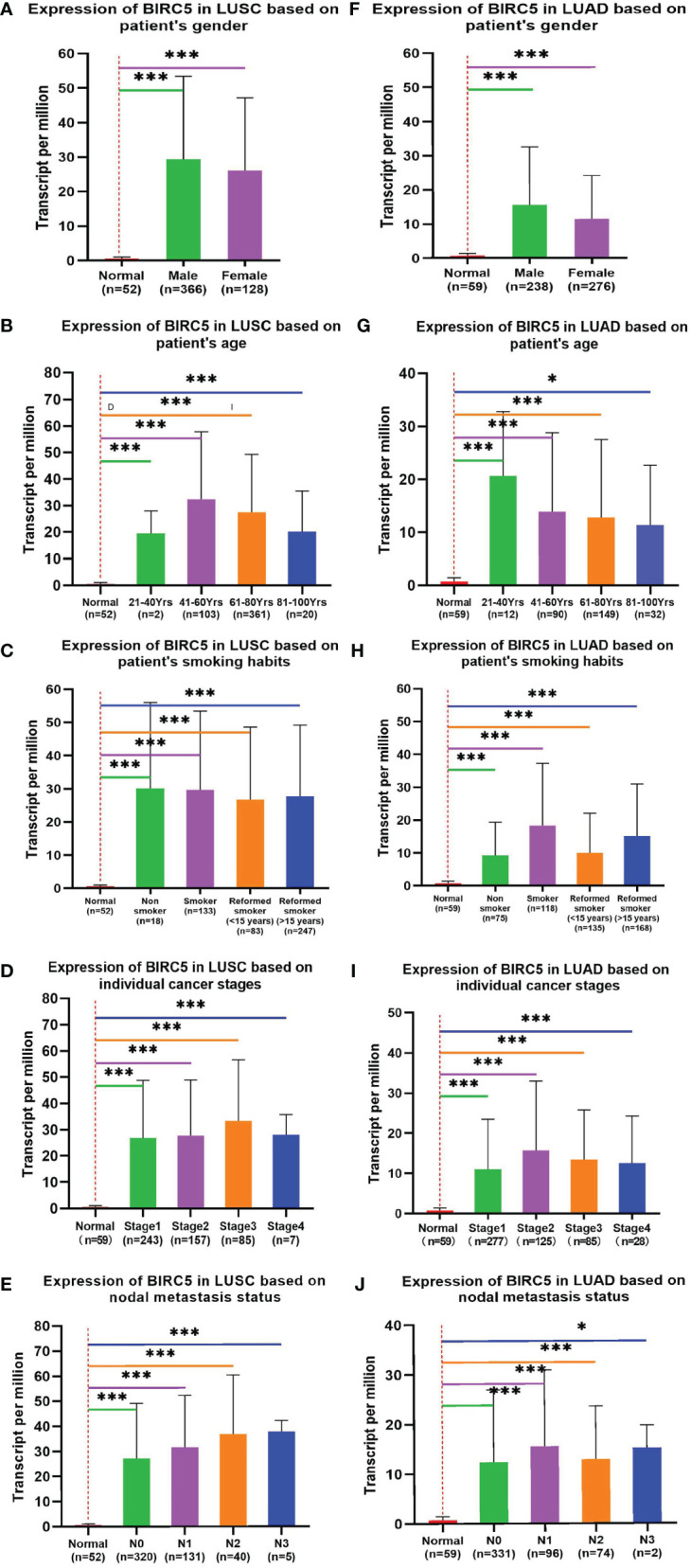
Correlation between BIRC5 expression and tumor stage in LUSC **(A–E)** and LUAD **(F–J)** patients in UALCAN; *p < 0.05, **p < 0.01, ***p < 0.001.

**Figure 7:**
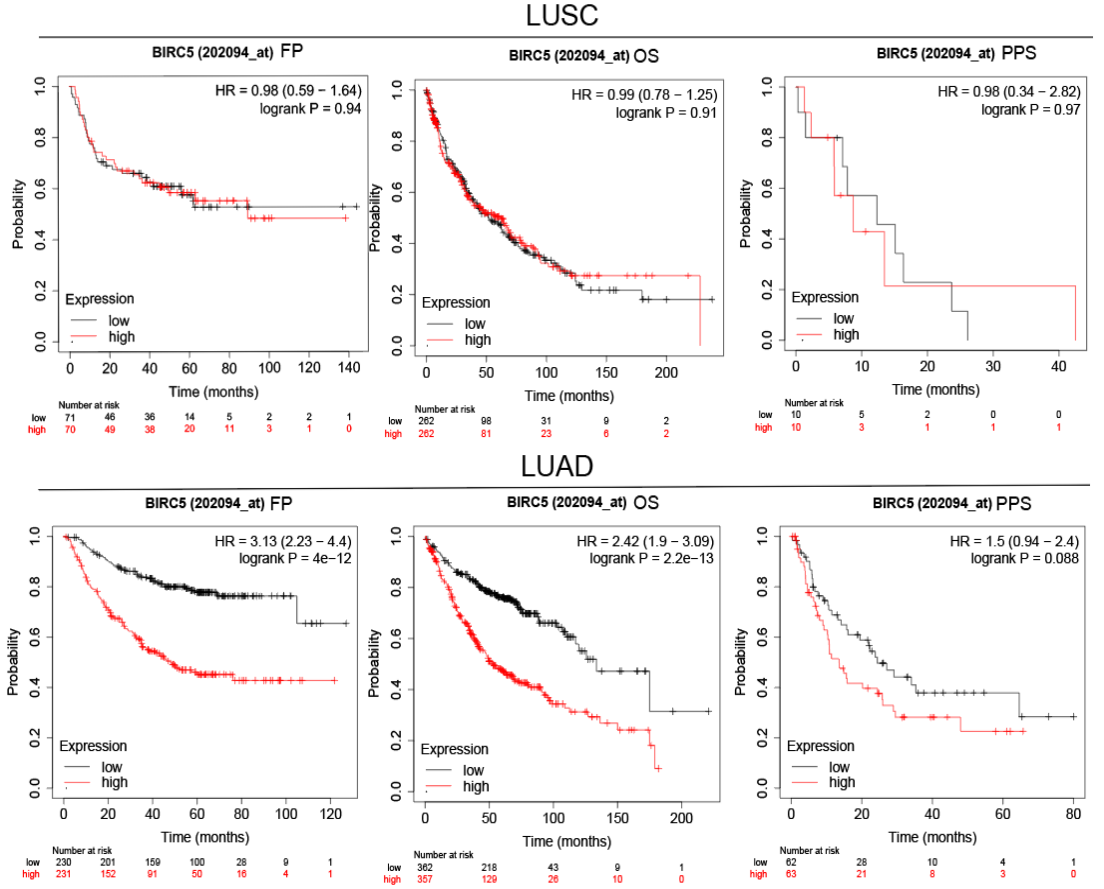
Survival analysis FP, OS and PPS of BIRC5 in LUSC and LUAD patients obtained from KM plotter.

To determine the role of BIRC5 in lung cancer, we first evaluated its expression and diagnostic and prognostic value in patients with NSCLC. Oncomine data revealed that mRNA expression of *BIRC5* was significantly higher in NSCLC tissues than in normal tissues (**Figures 5A, B**). According to the TCGA database, mRNA expression of BIRC5 in LUSC and LUAD tissues was higher than in normal tissues (**Figure 5C, D**). Using the human protein profile database, it was further verified that BIRC5 was positively expressed in NSCLC tissues and negatively expressed in normal lung tissues (**Figure 5E**). Then, we analyzed the transcription levels of BIRC5 by tumor stage, patients’ age, patients’ gender, smoking habits, and nodal metastasis status for LUSC and LUAD. Regardless of tumor stage, gender, age, smoking habits, and nodal metastasis status, BIRC5 transcription levels in tissues were significantly higher than in normal lung tissues (**Figure 6**). Furthermore, we investigated the correlation between BIRC5 overexpression at the mRNA level and patient prognosis by plotting and comparing FP, OS and PPS of LUSC and LUAD patients with that of healthy individuals through Kaplan-Meier plotter (**Figure 7**). BIRC5 overexpression was associated with worse FP (HR=3.13 (2.23-4.4), P<0.001), OS (HR=2.42 (1.9-3.09), P<0.001) in LUAD,which was negative in LUSC. Overall, the findings above imply that the mRNA expression of BIRC5 is remarkably correlated with LUAD patient survival rates, and BIRC5 expression may represent a promising biomarker for prediction of survival in LUAD patients. All of the above data indicate that the upregulation of BIRC5 expression levels promotes the development and progression of NSCLC.

### Relationship Between the Transcriptional Level of BIRC5 and Immune Infiltrates in LUSC AND LUAD

**Figure 8:**
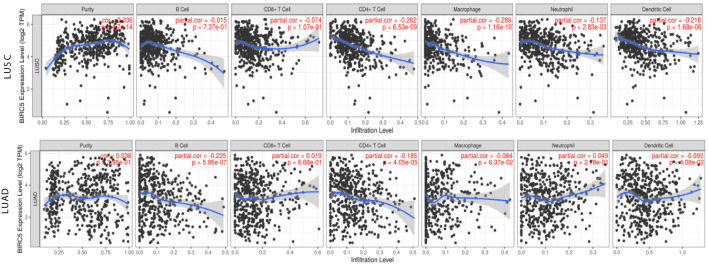
Relationship between the transcriptional level of BIRC5 and immune infiltrates in LUSC and LUAD.

Great progress has been made in immunotherapy for the treatment of advanced lung cancer (36). However, the role of BIRC5 in immune infiltrates in NSCLC is unknown. Using the TIMER database, we investigated the relationship between the transcriptional level of BIRC5 and immune infiltration. For LUSC, the levels of BIRC5 expression correlated positively with tumor purity (Cor=0.336, P=4.47e-14), while BIRC5 expression was negatively correlated with CD4+T cells (Cor=-0.262, P=6.53e-09, macrophages (Cor=-0.289, P=1.16e-10), neutrophils (Cor=-0.137, P=2.83e-03), and dendritic cells (Cor=-0.218, P=1.68e-06); there was no significant association between B cells and CD8+T cells. We also analyzed the distribution of immune infiltration in relation to LUAD mutated with BIRC5. The levels of BIRC5 expression correlated negatively with B cells (Cor=-0.225, P=5.95e-07), CD4+T cells (Cor=-0.185, P=4.05e-05), and dendritic cells (Cor=-0.093, P=4.08e-02), while there was no significant association between tumor purity, CD8+T cells, macrophages, neutrophils (**Figure 8**). The relationship between BIRC5 expression level and immune markers was analyzed. As shown in **Table 1**, the expression of BIRC5 correlated significantly with the expression of the marker genes of different subsets of T cells in LUAD, namely,B cell markers, CD19, CD79A;neutrophil markers CD66b (CEACAM8), CD11b (ITGAM), and CCR7;dendritic cell markers, HLA-DPB1, HLA-DQB1, HLA-DRA, HLA-DPA1, and BDCA-1 (CD1C); Th2 markers, STAT6 and STAT5A; Tfh markers, BCL6; Th17 markers, STAT3; Treg markers, STAT5B and TGFb (TGFB1); T cell exhaustion markers, LAG3 and GZMB. BIRC5 was negatively correlated with a variety of immune cells, suggesting that this gene is associated with immunotherapy resistance in NSCLC.

**Table 1:** Correlation analysis between BIRC5 and gene biomarkers of immune cells in LUAD (TIMER).

Immune cell	Biomarker	None		Purity	
		Cor	P-value	Cor	P-value
CD8+T cell	CD8A	0.124	4.86e-03	0.16	3.57e-04
	CD8B	0.176	5.89e-05	0.199	8.12e-06
T cell (general)	CD3D	0.008	8.59e-01	0.043	3.42e-01
	CD3E	-0.081	6.7e-02	-0.061	1.78e-01
B cell	CD2	-0.067	1.26e-01	-0.043	3.83e-01
	CD19	-0.125	4.4e-03	-0.121	7.33e-03
	CD79A	-0.13	3.21e-03	-0.124	5.75e-03
Monocyte	CD86	0.029	5.09e-01	0.059	1.90e-01
	CD115 (CSF1R)	-0.1	2.32e-02	-0.078	8.20e-02
TAM	CCL2	0.021	6.37e-01	0.049	2.74e-01
	CD68	0.0003	9.55e-01	0.023	0.04e-01
	IL10	0.017	7.02e-01	0.048	2.86e-01
M1 Macrophage	INOS (NOS2)	0.036	4.21e-01	0.026	5.71e-01
	IRF5	0.016	7.24e-01	0.034	4.52e-01
	COX2 (PTGS2)	0.058	1.88e-01	0.043	3.42e-01
M2 Macrophage	CD163	0.02	6.47e-01	0.044	3.35e-01
	VSIG4	-0.039	3.75e-01	-0.023	6.04e-01
	MS4A4A	-0.05	2.59e-01	-0.028	5.41e-01
Neutrophils	CD66b (CEACAM8)	-0.376	1.04e-18	-0.378	3.39e-18
	CD11b (ITGAM)	-0.153	5.04e-04	-0.133	3.12e-03
	CCR7	-0.233	9.66e-08	-0.236	1.21e-07
Natural killer cell	KIR2DL1	0.032	4.7e-01	0.036	4.26e-01
	KIR2DL3	0.176	6.15e-05	0.194	1.39e-05
	KIR2DL4	0.397	7.03e-21	0.421	1.20e-22
	KIR3DL1	0.063	1.5e-01	0.077	8.57e-02
	KIR3DL2	0.159	2.81e-04	0.191	2.05e-05
	KIR3DL3	0.157	3.46e-04	0.172	1.29e-04
	KIR2DS4	0.052	2.42e-01	0074	1.02e-01
Dendritic cell	HLA-DPB1	-0.367	6.64e-18	-0.375	7.02e-18
	HLA-DQB1	-0.287	3.13e-11	-0.285	1.20e-10
	HLA-DRA	-0.289	3.03e-11	-0.29	5.56e-11
	HLA-DPA1	-0.308	1.18e-12	-0.307	3.00e-12
	BDCA-1 (CD1C)	-0.485	9.52e-32	-0.482	5.32e-30
	BDCA-4 (NPR1)	-0.069	1.2e-01	-0.061	1.78e-01
	CD11C (ITGAX)	-0.067	1.31e-01	-0.05	2.67e-01
Th1	T-bet (TBX21)	-0.024	5.88e-01	-0.002	9.66e-01
	STAT4	-0.064	1.62e-01	-0.05	2.65e-01
	STAT1	0.349	3.74e-16	0.383	1.07e-18
	IFN-g (IFNG)	0.278	1.3e-10	0.317	6.13e-13
	TNF-α (TNF)	-0.039	3.74e-01	-0.006	9.00e-01
Th2	GATA3	0.004	9.35e-01	0.031	4.03e-01
	STAT6	-0.309	9.29e-13	-0.321	2.78e-13
	STAT5A	-0.124	4.81e-03	-0.104	2.1e-02
	IL13	-0.039	3.74e-01	-0.027	5.52e-01
Tfh	BCL6	-0.216	7.53e-07	-0.221	7.51e-07
Th17	STAT3	-0.153	5.06e-04	-0.162	3.09e-04
	IL17A	0.074	9.42e-02	0.081	7.24e-02
Treg	FOXP3	0.033	4.51e-01	0.061	1.73e-01
	CCR8	0.006	8.98e-01	0.032	4.72e-01
	STAT5B	-0.103	1.89e-02	-0.099	2.78e-02
	TGFb (TGFB1)	-0.164	1.93e-04	-0.155	5.41e-04
T cell exhaustion	PD-1 (PDCD1)	0.157	3.63e-04	0.203	5.53e-06
	CTLA4	0.065	1.42e-01	0.108	1.69e-02
	LAG3	0.232	1.1e-07	0.267	1.72e-09
	TIM-3 (HAVCR2)	0.028	5.19e-01	0.058	1.95e-01
	GZMB	0.407	5.44e-22	0.463	1.40e-27

### Enrichment Analysis of Co-Expression Genes Correlated With BIRC5 in LUAD

**Figure 9:**
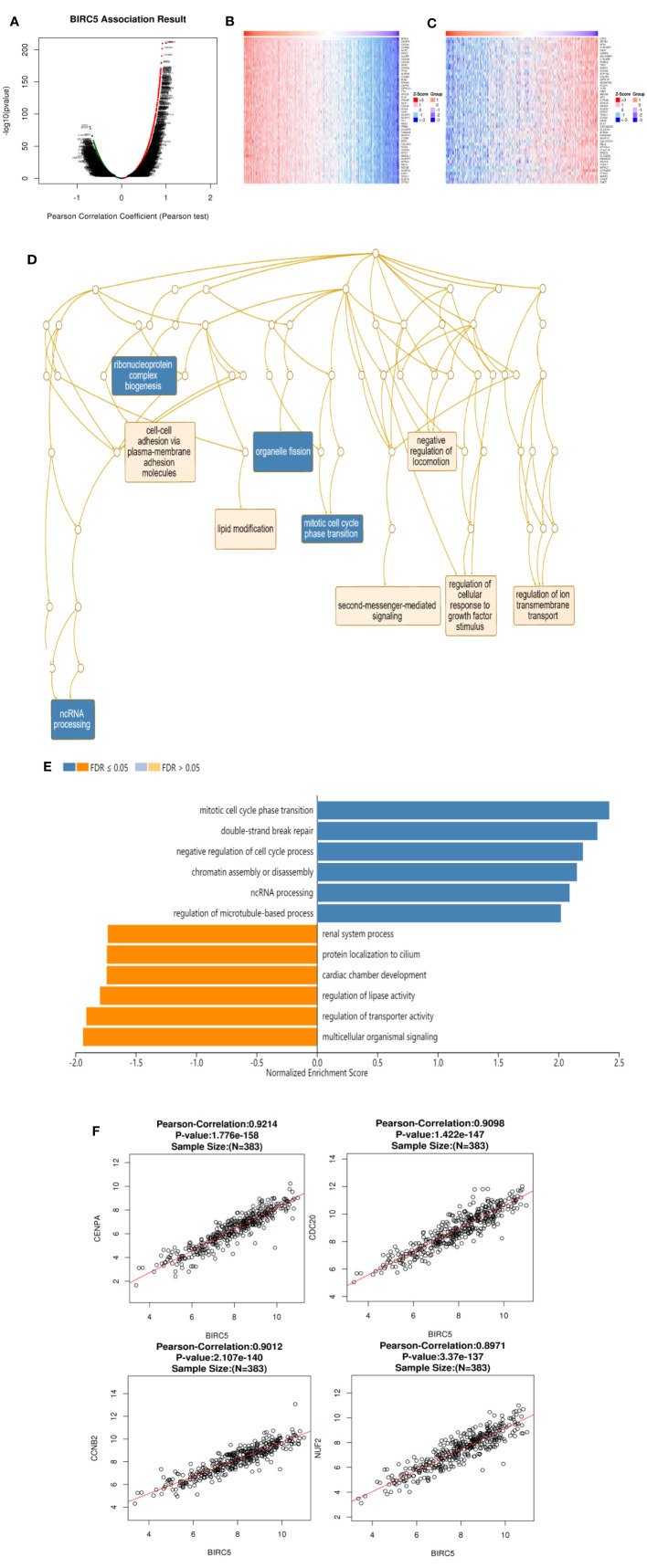
Genes co-expressed with BIRC5 in LUAD (LinkedOmics). **(A)** The genes positively and negatively correlated with BIRC5 in LUAD. Heat maps showing top 50 genes positively **(B)** and negatively **(C)** correlated with BIRC5 in LUAD. Red indicates positively correlated genes and blue indicates negatively correlated genes. **(D)** Significantly enriched GO annotations and KEGG pathways of BIRC5 in TCGA cohort **(E)**. The correlation between the hub genes and BIRC5 in LUAD **(F)**.

To gain insight into its biological significance, we examined BIRC5 co-expression and dark patterns, performed functional and enrichment analysis, and identified regulators of this gene in the LUAD cohort. The 5922 genes (red dots) showed positive correlations with BIRC5 and 7497 genes (green dots) showed negative correlations (**Figure 9A**). In addition, LinkedOmics was used to positively and negatively of the top 50 important genes associated with BIRC5 co-expression in LUAD (**Figures 9B, C**). Significant Gene Ontology (GO) term annotation indicated that BIRC5 correlated genes mainly participated in organelle fission, ribonucleoprotein complex biogenesis and mitotic cell cycle phase transition (**Figure 9D**). The KEGG pathway showed that BIRC5 related genes were primarily associated with mitotic cell cycle phase transition, double-strand break repair and negative regulation of cell cycle process (**Figure 9E**). Moreover, the significant top four genes were considered as the hub genes, namely CENPA (cor=9.214e-01, p=1.776e-158), CDC20 (cor=9.098e-01, p=1.422e-147), CCNB2 (cor=9.012e-01, p=2.107e-140), and NUF2 (cor=8.971e-01, p=3.370e-137); these were positively correlated with BIRC5 in LUAD (**Figure 9F**).

### The Networks of Kinase, miRNA or Transcription Factor Targets of BIRC5 in LUAD

Due to the significance of BIRC5 in LUAD, we further explored BIRC5 networks of kinase, miRNA or transcription factor targets in LUAD. For kinase networks of BIRC5, the top most significant targets were involved in the kinase AURKB. We found that the top 5 most significant miRNA network targets were MIR-17-5P, MIR-507, MIR-24, MIR-138 and MIR-199A (**Table 2**). Moreover, the transcription factor network target (V$E2F1_Q6, V$E2F4DP1_01, V$E2F_Q6, V$E2F_Q4, V$E2F_Q4_01) of BIRC5 was shown in **Table 2**.

**Table 2:** The Kinase, miRNA and transcription factor-target networks of BIRC5 in LUAD (LinkedOmics).

Enriched Category	Geneset	LeadingEdgeNum	FDR	P
Kinase Target	Kinase_AURKB	11	0	0
miRNA Target	GCACTTT, MIR-17-5P	65	0.401	0
	GTGCAAA, MIR-507	28	0.372	0.019
	CTGAGCC, MIR-24	27	0.394	0.006
	CACCAGC, MIR-138	17	0.337	0.005
	ACACTGG, MIR-199A	14	0.353	0.005
Transcription Factor Target	V$E2F1_Q6	81	0	0
	V$E2F4DP1_01	80	0	0
	V$E2F_Q6	80	0	0
	V$E2F_Q4	80	0	0
	V$E2F_Q4_01	74	0	0

### Prediction of the Resistance Mechanism of BIRC5 in NSCLC

**Figure 10:**
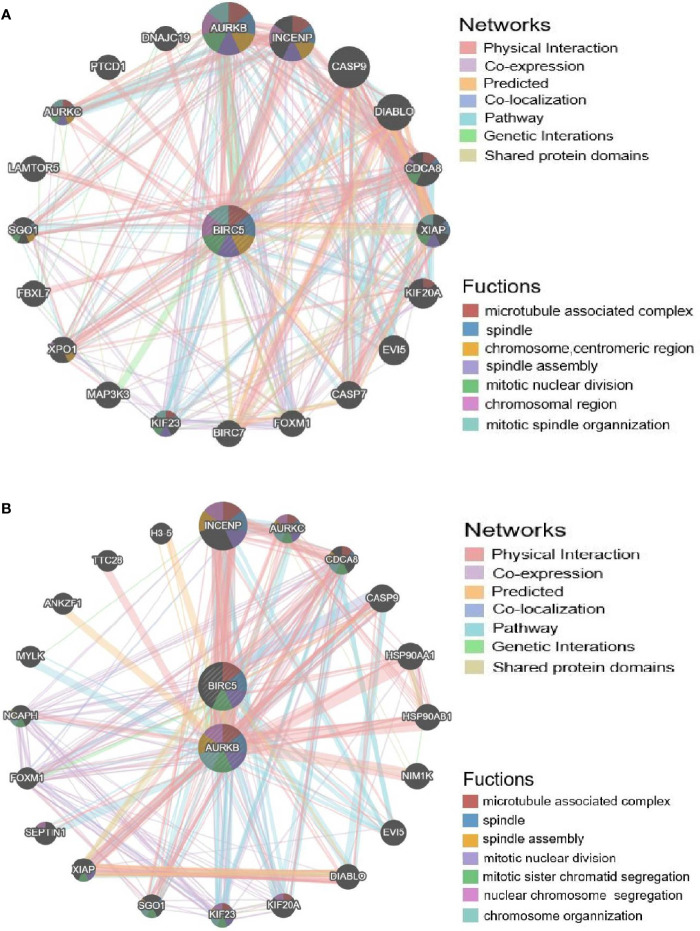
Validation of the afatinib resistance function of BIRC5. **(A)** PPI network of BIRC5 produced by GeneMANIA. **(B)** PPI network of BIRC5 and AURKB.

To determine whether BIRC5 functions in the drug resistance of cancer, GeneMANIA was employed. BIRC5 showed interactions with 20 proteins/genes. And the results showed that these genes were primarily involved in the regulation of microtubule associated complex, spindle, spindle assembly, chromosome, centromeric region, mitotic nuclear division, chromosomal region and mitotic spindle organization (**Figure 10A**).

To further explore the regulators of BIRC5 in expression, we performed an analysis of kinases, miRNAs and transcription factors of BIRC5 gene (**Table 2**). For kinase networks of BIRC5, only one kinase target of BIRC5 was identified (Kinase_AURKB) according to the LinkedOmics database. To probe the function of AURKB in more details, we next constructed PPI networks of AURKB. And the results showed that these genes were primarily involved in the regulation of microtubule associated complex, spindle, spindle assembly and mitotic nuclear division (**Figure 10B**).

### Genetic Pathway and Drug Sensitivity Analysis of Hub Genes

**Figure 11:**
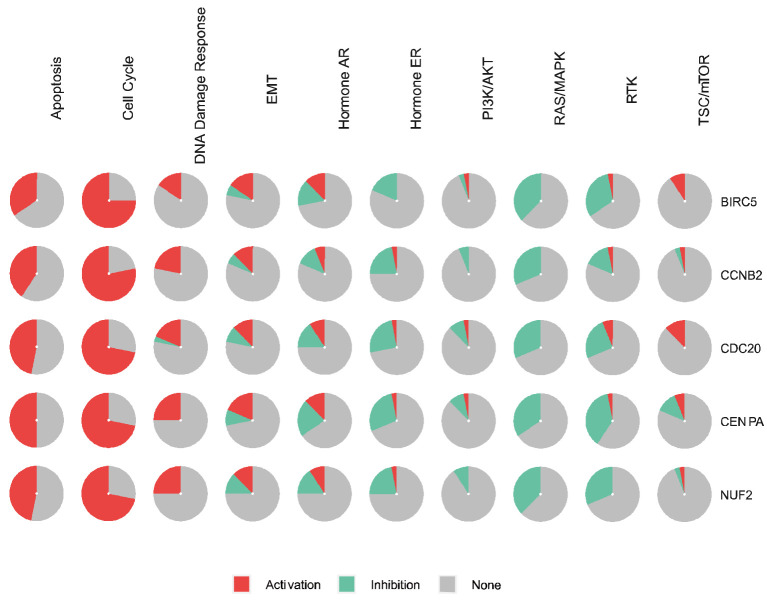
The role of BIRC5 and top four genes in the cancer-related pathways (GSCALite).

BIRC5 and the top four significant genes, namely *CENPA, CDC20, CCNB2*, and *NUF2*, were selected as the hub genes for genetic alteration, pathway, and drug sensitivity analyses. We also explored the role of hub genes in all known cancer related pathways, including Apoptosis, Cell Cycle, DNA Damage Response, EMT, Hormone AR, Hormone ER, PI3K/AKT, RAS/MAPK, RTK, and TSC/mTOR pathways. Results showed that BIRC5 is involved in the activation of Apoptosis, Cell Cycle, DNA Damage Response, EMT, and Hormone AR pathways (**Figure 11**).

**Figure 12:**
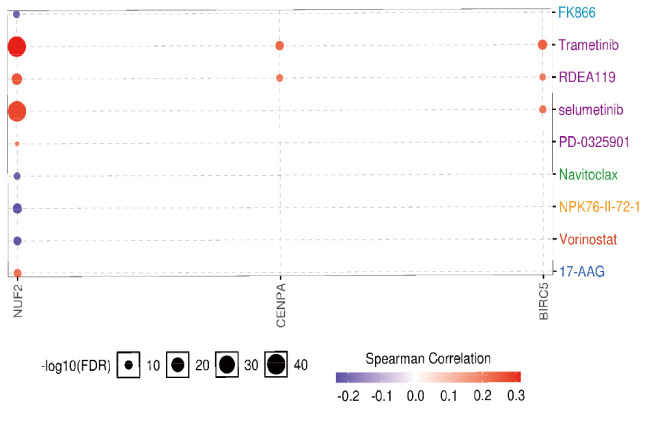
The drug resistance analysis of hub genes based on GDSC IC50 drug data. Correlation between gene expression and the drug is represented by Spearman correlation, and positive correlation means that high expression is associated with drug resistance.

By drug sensitivity analysis, we evaluated the correlation of gene expression and IC50 using Genomics of Drug Sensitivity in Cancer (GDSC) of small molecules. As shown in **Figure 12**, a high NUF2 level was resistant to 5 drugs or small molecules, whereas a high CENPA level was resistant to 2 drugs or small molecules. Moreover, high expression of BIRC5 was associated with resistance to trametinib, RDEA119, and selumetinib. These findings may provide support for the selection of drugs for targeted therapy in LUAD.

### Upregulation of BIRC5 Correlates With Afatinib Resistance

**Figure 13:**
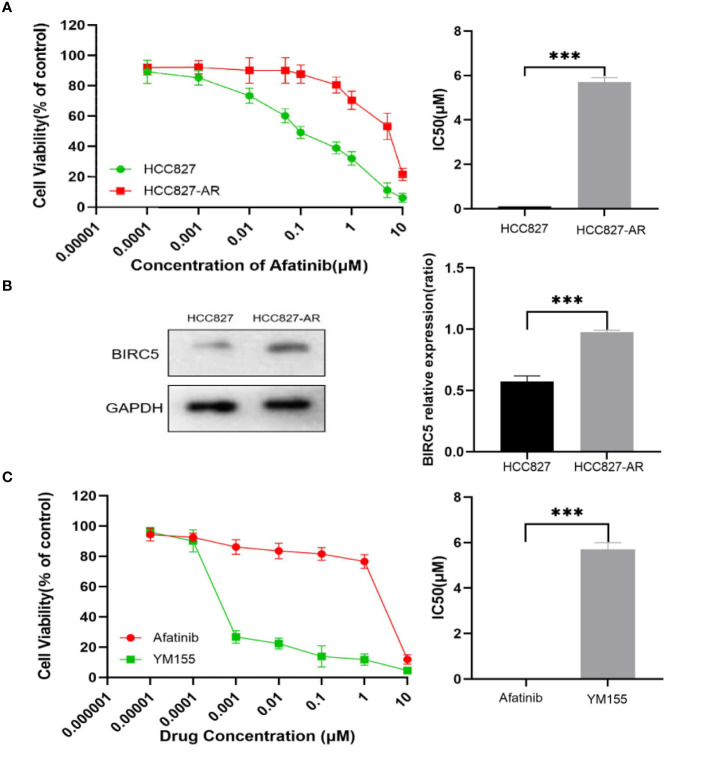
Induce and detect HCC827 and HCC827-AR cells. **(A)** HCC827 and HCC827-AR cells of cell viability assays are shown. **(B)** Protein expression levels of BIRC5 in HCC827 and HCC827-AR cells. **(C)** Effects of afatinib and YM155 on HCC827-AR cells as observed via cell viability assays are shown. ***p < 0.001.

In the present study, stepwise exposure to increasing concentrations of afatinib was used to establish afatinib-resistant lung cancer cells. The half maximal inhibitory concentration (IC50) of afatinib treated for 72 h in HCC827 and HCC827-AR cells was measured by CCK8 (**Figure 13A**). To explore the role of BIRC5 in NSCLC cell resistance to afatinib, western blot analysis was performed. As shown in **Figure 13B**, BIRC5 expression was significantly higher in HCC827-AR cells than in HCC827 cells (P<0.001). Next, we tested the sensitivity of HCC827-AR cells to afatinib and BIRC5 inhibitor YM155 *in vitro*. HCC827-AR has a half-maximal inhibitory concentration of afatinib (IC50)=1–10 μM), YM155 (IC 50 = 1–10 nM) (**Figure 13C**). The results confirmed that YM155 is about 1000 times more potent than afatinib *in vitro* in afatinib-resistant cells. This finding provides potential new therapeutic strategies against lung adenocarcinoma cells with acquired drug resistance.

## Discussion

Over three decades, a mainstay and goal of clinical oncology were the development of therapies that promote the effective elimination of cancer cells by apoptosis (37). The evasion of apoptosis is a common strategy adopted by cancer cells for drug resistance. BIRC5, a member of the human inhibitors of apoptosis proteins (IAPs) family is one of the most studied molecular and therapeutic targets in various type of cancer *s* (38, 39). Aberrant expression of BIRC5 in cancers have been found to facilitate cancer progression (40, 41).

In the current study, we first focused on the expression features value of BIRC5 in afatinib-resistant lung cancer. From GEO data, we selected DEGs of the number of afatinib-resistant cells, lung cancer and normal tissues, and selected BIRC5 for further research. As elucidated in the GO and KEGG analyses of DEGs, the nodules in this network were enriched in mitosis and cell cycle pathways (**Figures 1**–**4**). Actually, BIRC5 is a key regulator for apoptosis and mitotic spindle checkpoint. Overexpression of BIRC5 usually result in aberrant mitosis of transformed cells. Cancer cells exit from drug-induced mitotic slippage to avoid subsequent cell death which is a major mechanism contributing to resistance (42). Overexpression of BIRC5 was observed in about half of the pan-cancers in the Oncomine database, and NSCLC showed the greatest difference between normal and tumor tissues. In order to further confirm the clinical value of BIRC5 expression in the diagnosis of lung cancer, we further analyzed TCGA dataset, and the results were shown to be consistent with the observations in the Oncomine data. Overexpression of BIRC5 in NSCLC tissues was confirmed (**Figure 5**).

We further focus on LUAD and LUSC to discover the significance of BIRC5 and explore the clinical significance and potential function of BIRC5. Herein, we found that the overexpression of BIRC5 was significantly correlated with tumor stage, age, gender, smoking habits, and nodal metastasis status in normal tissues **(** **Figure 6**). BIRC5 also performed well in predicting the overall survival of LUAD patients in the TCGA data (**Figure 7**). Similarly, BIRC5 is associated with progression and poor survival in LUAD, linking overexpression of BIRC5 with an increased invasive phenotype and worse clinical prognosis.

According to the analysis in TIMER, aberrant expression of BIRC5 may alter cancer microenvironment and immune response, thus impact on the overall clinical outcome. We confirmed that overexpression of BIRC5 was associated with decreased immune cells infiltration in LUAD (**Figure 8**). High expression levels of BIRC5 was observed in tumor-adjacent immune cells (43). Taken together, these results suggest that the abnormal expression of BIRC5 may contribute to the poor effect of immunotherapy.

Through coexistence analysis, we found that BIRC5 and its related genes are involved in the cell cycle regulation and DNA replication pathway, as well as related to the expression of *CENPA, CDC20, CCNB2* and *NUF2* genes. Similarly, as elucidated by lung cancer-specific PPI, the nodules in this network are rich in processes and pathways related to apoptosis, cell cycle and DNA damage. Functional analysis of BIRC5 protein network revealed that several kinases closely interact with BIRC5 including AURKB. AURKB is a kinase that plays an important role in cell division and aberrant activation of AURKB was found in NSCLC (44). BIRC5 is a multifunctional gene that can function as inhibitor of cell death and essential regulator for cell mitosis (45). An increased tendency of BIRC5 during the life cycle of cells may lead to genomic instability, which is a major driving force for tumorigenesis (46). In addition, the increase of BIRC5 indicates multi-drug resistance in various type of cancer. Finally, by establishing an afatinib-resistant lung cancer cell line *in vitro*, HCC827-AR, we further confirmed that BIRC5 was overexpressed in lung adenocarcinoma cell line afatinib resistant cells at the protein levels. The BIRC5 inhibitor, YM155, showed an inhibitory effect on the cells resistant to afatinib. Silencing of BIRC5 also caused proliferation inhibition and induced apoptosis in lung cancer cells (47). Recently, it has been reported that combination of BIRC5 inhibitor with osimertinib can effectively inhibit the growth of lung cancer in mouse xenograft model (48), and a phase II clinical trial showed YM155 exhibited modest single-agent activity in patients with refractory in advanced NSCLC (49), indicating that BIRC5 may be a new target to overcome TKI resistance in LUAD.

Despite of the promising performance of the BIRC5 signature, there were certain limitations for the current study. The establishment and validation of the research were based on the public sequence data. Moreover, the DEGs in the article are considered too loose for the significant p value threshold for microarray analysis to apply. Further functional validation of BIRC5 may shed new light on mechanisms of afatinib-resistant in lung cancer, such as more details of mechanisms and signaling network involved in TKI resistance in LUAD as well as multi-center clinical trials of targeting BIRC5 in combination with TKIs or immunotherapy in NSCLC patients.

## Conclusion

In conclusion, the current study mainly focuses on the investigation of the expression features and potential functions of BIRC5 in NSCLC. We have demonstrated that overexpression of BIRC5 resulted in resistance to afatinib in NSCLC, and BIRC5-specific inhibitors may reverse the resistant phenotype and promote cell death of lung cancer cells. Our study provided evidence for the future investigation of BIRC5 as new target for overcoming TKI resistance and the prognostic value for NSCLC.

## Data Availability Statement

The datasets presented in this study can be found in online repositories. The names of the repositories and accession numbers can be found in the article material.

## Author Contributions

XZ: Conceptualization, Formal analysis, drafting manuscript, review & editing. QC and YZ: Data curation. RZ and YL: proof editing. YZL: Conceptualization,proof editing, funding support. All authors contributed to the article and approved the submitted version.

## Funding

This study was supported by a grant from the National Natural Science Foundation of China (Grant No. 81572606).

## Conflict of Interest

The authors declare that the research was conducted in the absence of any commercial or financial relationships that could be construed as a potential conflict of interest.

## Publisher’s Note

All claims expressed in this article are solely those of the authors and do not necessarily represent those of their affiliated organizations, or those of the publisher, the editors and the reviewers. Any product that may be evaluated in this article, or claim that may be made by its manufacturer, is not guaranteed or endorsed by the publisher.
